# Digital selection of composite resin shade using cross polarized photography and a standardized white balance gray reference card

**DOI:** 10.4317/jced.58340

**Published:** 2021-10-01

**Authors:** Panagiotis Ntovas, Efstratios Papazoglou

**Affiliations:** 1 DDS, Postgraduate Student, Department of Operative Dentistry University of Athens, Greece; 2 DDS, MSc, PhD, Associate Professor, Department of Operative Dentistry, University of Athens, Greece

## Abstract

**Purpose:**

The aim of the present case report was to describe, through a clinical case, a step-by-step technique, for the digital selection of composite resin shades using cross polarization and white balance employing a standardized reference card.

**Case Description:**

After intraoral impression, a digital diagnostic wax up was developed. A 3D printed cast was used to fabricate a lingual polyvinyl siloxane matrix. First a cross-polarized image of the contralateral central incisor was taken in RAW format, using a gray reference card. The digital image was manipulated using this card as a reference, to obtain reliable color values. The obtained color values, from the contralateral central incisor, were used in order to digitally find the closest combination of enamel and dentin shades between various composite systems. The values of a combination of shades of composite systems, was captured using cross-polarized images, with a gray reference card, after digital image manipulation of composite specimens, which have been created for this purpose.

**Conclusions:**

Digital evaluation and selection of composite resin system and shade, using a white balance reference card assisted by cross polarized photography, could help achieving more predictable outcomes using the stratification technique with composites.

** Key words:**Restoration shade, composite color, composite resin, direct restoration, digital protocol.

## Introduction

In anterior direct restorations, the replication of the optical characteristics of natural teeth constitutes a challenge (1). Better understanding of the inherent characteristics of the dental tissues, allied with the improvement of restorative techniques, grant the opportunity for the construction of natural looking restorations (2). However, shade selection with composite resins can still be a challenge.

Shade selection usually is carried out subjectively, using prefabricated shade guides under non-controlled light conditions, leading to errors in color selection (3). These shade guides often are made from ceramic with different optical properties compared to composite resins (4). Also, there are differences in composite’s color, for the same shade among commercial brands or even batch numbers.

Application and light curing of composite resin masses (buttons) directly on the corresponding regions of a sound tooth’s surface, might be a reliable technique for shade selection (3,5). However, with this technique the influence of the stratification and composite’s color change due to hydration cannot be estimated (6). Construction of custom-made shade guides with different composite shade combinations in various thicknesses, can be a viable option. However, these shade guides are prone to color change over time. Nonetheless, these techniques remain subjective, as shade selection is still highly dependent on visual perception, which depends on the clinical experience of the operator (3,7).

Digital technology has introduced new possibilities in color analysis. Special devices for color measurement, namely colorimeters and spectrophotometers, aim to reduce subjective color selection, resulting in a more predicTable color matching, even for inexperienced operator (8,9). But many of these devices have a high cost, can indicate an incorrect color and may not be compatible with resin composites, since most of them pertain to ceramic restorations (8,10).

Photography contains a wealth of color information, but does not provide consistent color accuracy, as color in digital images is influenced by technical factors, such as lighting, output medium, camera, capture settings etc (11). Recently, there has been an increasing interest in illumination techniques. The use of images captured under cross polarization, be means of placing one polarizing filter in front of the lens and one in front of the light source with a 90o rotation to the other, can assist in better evaluation of the color characteristics, minimizing specular reflection and errors during the shade matching of natural teeth or restorations. This technique was first described for diagnostic purposes in dentistry and as an aid in color selection, and, translucency identification using analogue SLR cameras (12-14). Later, use of polarized photography was described for shade selection with digital cameras (dSLRs) (15). This technique allows the unobstructed visualization of surface and subsurface enamel characteristics, by eliminating the superficial glare which removes all color information (16-18).

Furthermore, the use of cross polarization along with a standardized white balance gray reference card, as a known reference, can achieve image standardization, allowing a more objective image analysis and a numerical quantification of the color (10,19,20). Hein and *et al*. (10) used cross polarization to photograph the dentin and enamel ceramic masses for the most common ceramic systems achieving a library of CIE L*a*b* color coordinates by means of a computer software. The coordinates of the library were matched with the coordinates derived by the clinical photograph of the tooth, giving the closest match in color ceramic masses (10). However, there is not a comprehensive protocol in the literature describing the application of the aforementioned technique, to direct composite resin restorations.

The purpose of the present case report was to describe, through a clinical case, a step-by-step technique, for the digital selection of composite resin shades using cross polarization and white balance employing a standardized reference card.

## Case Report

A 19-year-old female patient was referred for treatment, expressing dissatisfaction about with the appearance of her smile. Her dental history revealed that she had her maxillary left central incisor restored multiple times with composite, after a dental trauma during a basketball game before 10 years. Previous composite resin restorations did not match the contralateral tooth in color, contour or texture. The tooth responded within normal limits to cold pulp testing, without evidence of periapical radiolucency or calcified root canal. Patient’s periodontium was healthy. A decision to replace the direct composite resin restoration with a new one was made, after analyzing the appropriate restorative options, the benefits, drawbacks and costs with the patient.

Treatment was initiated with 3 weeks of home bleaching, using a 10% carbamide peroxide gel, (Opalescence 10%; Ultradent Products, Inc, South Jordan, USA), with a bleaching tray without reservoirs (10). A digital diagnostic wax up was developed (3Shape LabStudio, Copenhagen, Denmark), after intraoral impression (Trios 4, 3Shape, Copenhagen, Denmark). A 3D printed cast was used to fabricate a lingual polyvinyl siloxane (PVS; Reprosil VPS Impression Material Putty; Dentsply Sirona, Bensheim, Germany) matrix.

Twenty days after the end of the home bleaching, shade selection was performed. First a cross-polarized image of the contralateral central incisor in RAW format was taken using a DSLR Camera (80D, Canon, Tokyo, Japan), equipped with a 100mm lens (EF 100mm, f/2.8, IS USM, Canon, Tokyo, Japan) and a Prototype cross-polarization filter, attached to a twin flash system (270EX II, Canon, Tokyo, Japan). An aluminum plate with a thickness of 3mm with a circular hole in front of the lens and two rectangular holes in from of the flash area was designed and milled. In these holes linear polarization film sheets were attached, with the lens sheet perpendicular to the flash sheets. A gray reference card (white balance, Emulation, Freiburg, Germany), was placed under the central incisors. The following camera settings were used: shutter speed 1/125sec, aperture f22, sensor sensitivity ISO 100, ½ of flash power and 1:2.5 magnification ratio.

**Figure 1:**
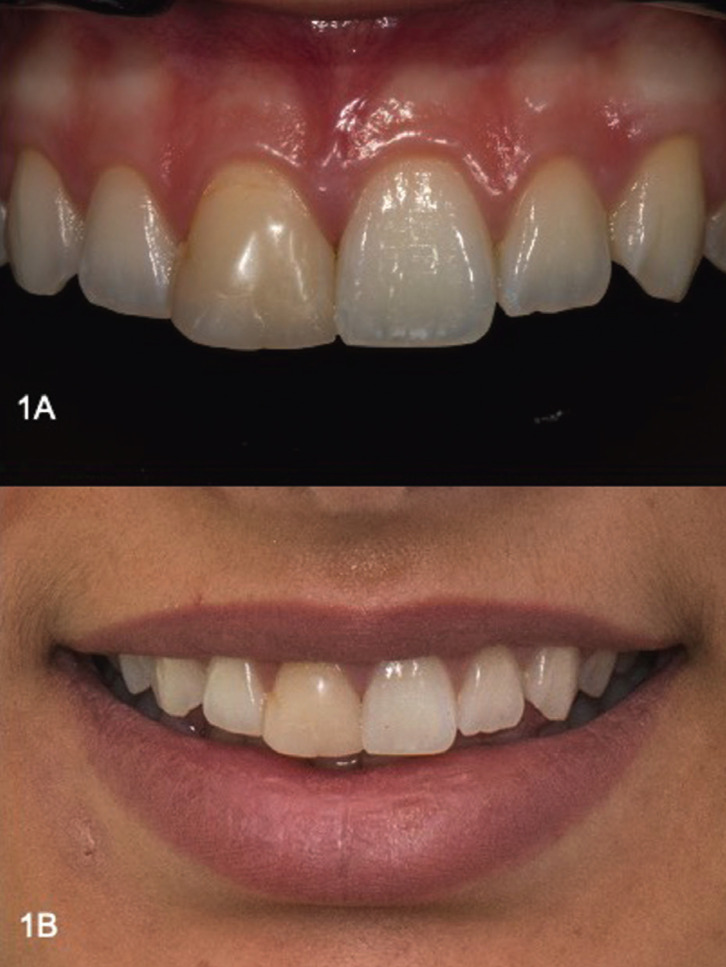
Initial Situation A) Close up, B) Smile.

**Figure 2:**
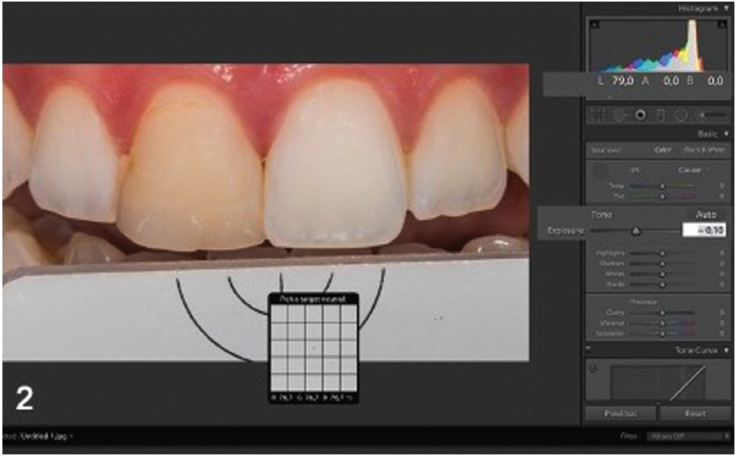
Customized white and exposure balance in Lightroom software using the reference card.

The digital image was manipulated using an image editing software (Lightroom CC, Adobe, California, USA). At first, the appropriate camera’s profile was selected, before the white and exposure balance. The white balance was performed by clicking with the special tool of the software to the part of the gray reference card (White balance, Emulation, Freiburg, Germany), under the right central incisor (Fig. 1). To carry out exposure balance, exposure of the image was manipulated via the software Lightroom (Adobe, California, USA), until the color value parameters (L, a, b) of the reference card in the image, matched with the real values of L, a and b of the reference card which are 79, 0, 0 (Fig. 2).

After the white balance of the image, a color meter software (Digital Color Meter, California, USA) was used with an iOS operating laptop (MacBook Pro, Apple, California, USA to obtain tooth’s color coordinates in CIE Lab color space. The obtained color values, from the contralateral central incisor, were used in order to digitally find the closest combination of enamel and dentin shades of the available to our clinic composite systems. The values of our case were inserted in a spreadsheet (Excel, Microsoft, New Mexico, USA). This file contained the data of the available to our clinic composite resin systems, for each shade of enamel and dentin alone and all possible enamel-dentin combinations. To obtain these data, specimens of dentin shades for various systems of composite were constructed, in a thickness of 2 mm, using a metallic cylindrical mold (Porcelain Sampler, SmileLine, St-Imier, Switzerland). In the same way, specimens of enamel shades for 2 different thicknesses of enamel (0.5mm and 0.9mm) were created. Photographs of all of these samples were taken, individually for each specimen and for every possible dentin-enamel combination for each composite system. Specimens along with the gray reference card (white balance, Emulation), were placed in a transparent glass, 12 cm away from a black background, utilizing the same camera settings as in intraoral photographs. Glycerin gel was applied between enamel and dentin specimens to ensure optical continuity. These data were collected using cross-polarized images, with a gray reference card, after digital image manipulation, in the same way and with the same settings, as in the procedure which was described previously in our case. In the excel the following color difference formula ΔΕ= [(ΔL)2 + (Δa)2 + (Δb)2]½, was used between the numerical color data received from the contralateral central incisor and the data from the composite disks on file. This wat the closest combination of enamel and dentin composite that would match the contralateral incisor was found.

**Figure 3:**
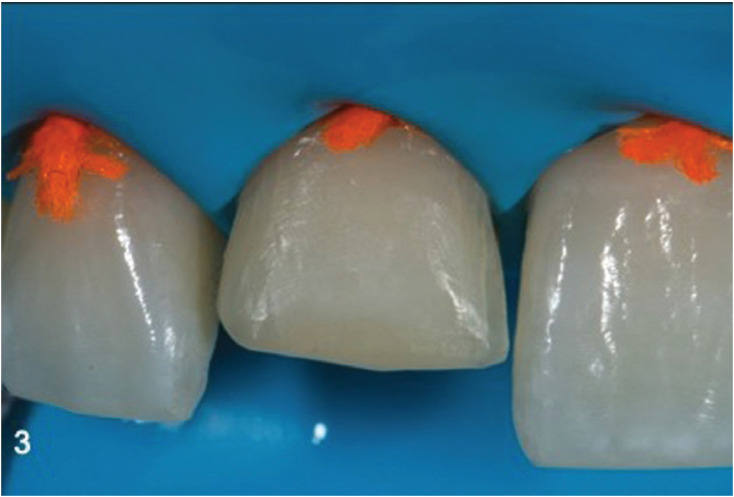
After the remove of old restoration.

Rubber dam isolation after local anesthesia was established. A 1.5-mm functional-esthetic enamel bevel was prepared on the facial using a fine diamond bur (886 314 012, Komet, Lemgo, Germany). A functional bevel of 1mm was performed lingually in a chamfer finish line conFiguration. The facial bevel was extended interproximally and towards the gingival third using a coarse disc (Sof-lex, 3M ESPE, Seefeld, Germany), to create a “infinite bevel” in order to achieve indistinguishable restoration margins (Fig. 3). Polytetrafluoroethylene tape was placed on the adjacent teeth to protect them from the application of 35% phosphoric acid (Scotchbond,3M ESPE) to the enamel for 30s. After rinsing with water, adhesive (Adhese Universal, Vivapen, Ivoclar Vivadent, Schaan Liechtenstein) was applied and light activated for 20s (Bluephase G4, Ivoclar Vivadent).

**Figure 4:**
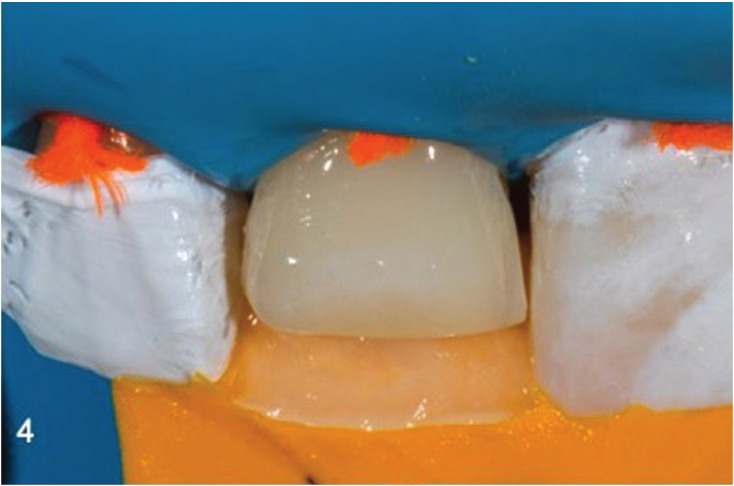
Application of a lingual layer of enamel.

**Figure 5:**
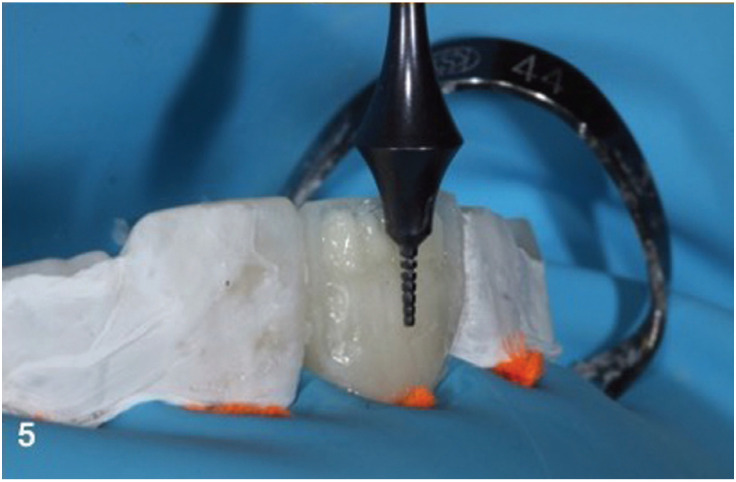
A special caliper (TNFF7/8, Hu Friedy) was used to control the thickness of dentinal material based to the thickness of our measurements.

The lingual PVS matrix was loaded with the selected enamel shade (Skin White Enamel InSpiro, EdelweissDR, Zug, Switzerland) and seated, with finger pressure (Fig. 4). After light curing the PVS matrix and Teflon tape were removed. A sectional metal contoured matrix was placed to restore the interproximal walls and contacts (TorVm, Moscow, Russian). Dentin shade (i1 Body, In Spiro, EdelweissDR) was shaped extending from the middle of the bevel area to the incisal edge, forming incisal edge inner anatomy. In order to determine the thickness of residual buccal enamel, according to our initial color measurements, a special caliper (TNFF7/8, Chicago, Hu Friedy) was used (Fig. 5). Finally, an enamel layer of 0.9mm thickness was applied, extending from the beveled areas to the incisal edge.

**Figure 6:**
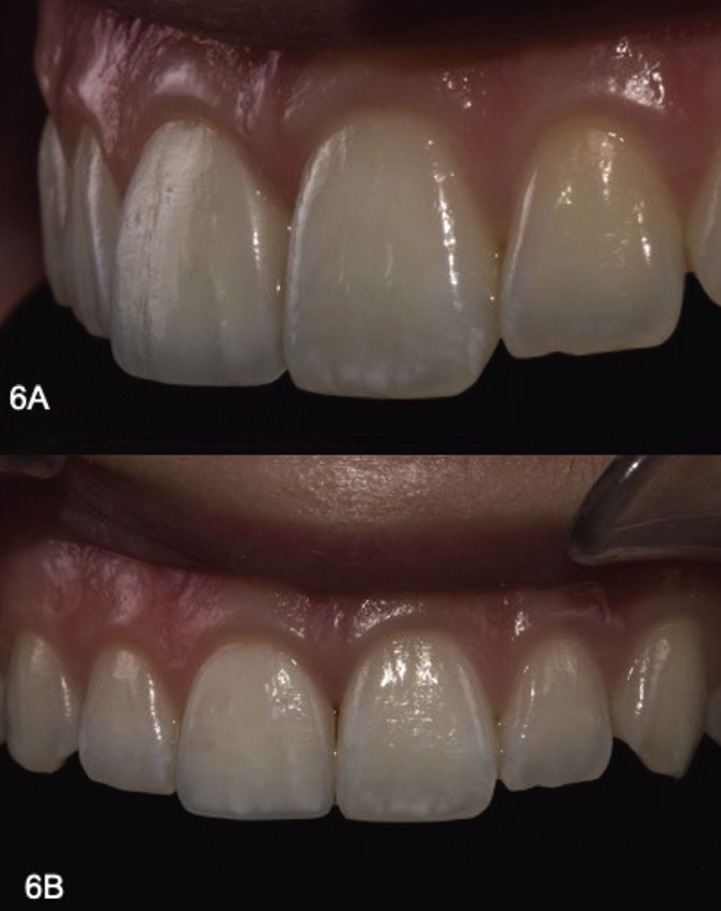
Final Outcome A) Close Up B) Smile.

Line angels of both central incisors were highlighted using a mechanical pencil. Finishing of the restoration was accomplished, using coarse and medium disks (Sof-Lex 3M ESPE) , a low speed flame diamond bur (831 204 012, Komet, Lemgo, Germany) for smoothening and shape conFiguration, a #12 surgical blade (Swan-Morton, Sheffield, UK) for removing excess in proximal areas, a fine multi-blade carbide bur for fine adjustments in labial surface (H48LUF 314 012, Komet) and a high speed medium grit diamond football bur (8379 314 021, Komet), for palatal occlusal adjustments. A fine grit diamond bur (864 014 12F, Komet) was also used to create micro-texture. Restoration was polished using a fine and extra fine polishing discs (Sof-Lex 3M, ESPE) from medium to superfine for marginal ridges, and special polishers (Eve Diacomp Plus Twist polishers, Stuttgart, Germany) for the facial and lingual. Final luster was achieved using a system of diamond and silicon carbide-based pastes (Enamel Plus Hfo Polishing Paste Kit, Micerium, Genova, Italy) applied by 3 different goat hair brushes and a felt wheel without paste at the end. Final esthetic evaluation, including shade and texture, was performed 14 days postoperatively (Fig. 6).

## Discussion

Resin composites are the norm in the conservative restoration of a single anterior incisor. The use of shade guides remains a complex process.4 Shade selection involves subjective factors that depend directly on the observer, the light source, and reflection of light (22,23). Moreover, in direct restorations, the selection of composite resins with various optical properties is important in order to imitate enamel and dentin via the layering procedure (23,24).

Special color measurement instruments are owned by a small percentage of clinicians. Digital camera with the use of proprietary software has been considered an alternative method to the colorimeters and spectrophotometers, in assessing color (10,23,24). Instrumental assessment of color is not free of errors, as teeth are translucent, polychromatic with curved surfaces (26). Digital image allows also, a better understanding of the details, characteristics depth and transparency of the dental structure, as they can be enlarged and manipulated in various aspects (16,17,27). All this information can be evaluated and used in order to optimize the stratification technique with resin composites, minimizing the errors in clinical practice (23,27,28).

Until now dSLRs or mirrorless cameras seem to be the adequate equipment for the proposed technique described in the article. Mobile phones can be a significant less expensive alternative in every day clinical practice, especially with additional accessory light equipment (29). However, many mobile phones cannot shoot in RAW format, which is important for the further manipulation of the images without losing their quality, as software interpretation seems to play a significant role (18,19,30).

The use of cross-polarization filter along with a white balance card with known color coordinates, seems to improve the evaluation of shade for resin composites restorations, reducing the effect of the photographic equipment and environmental light (18,19). However, the proper digital camera settings, illumination conditions and object-camera distance have to be standardized as much as possible in order to take the most accurate color values (10,19). Construction of custom-made tooth-shaped shade guides with different composite shade combinations in various thicknesses, is a viable alternative to the disk shaped specimens. Comparing this technique to the use of custom-made composite shade guides, the selection is made objectively using numerical data compared to visual perception and there is not a need for every clinician to make the guides individually. Additionally, these shade guides may present color change over time.

By the proposed technique, the clinician can choose the proper combination of enamel and dentin shades, which will lead to the closest match, giving a great chance of meeting patient expectations. The subjectivity in color selection is minimized as color selection is based on digital quantification. Additionally, digital images offer all the information the clinician needs to mimic the anatomy during stratification. The technique presented is a tool for their inspection and verification of the final result. Image capture, its further process and the planning of the restoration with the proposed technique require few additional minutes, in which a blueprint of the restoration is designed. Another advantage with the proposed technique is that specimens before image capture were kept in distilled water for 2 weeks. Therefore, initial color change of the composited due to hydration was also taken in consideration during shade selection (6).

The accuracy of the presented protocol has to be further investigated. The influence in color difference of the geometry between the flat composite specimens which was used in order to obtain the color values of the composites and the curved tooth, has to be evaluated. Color matching of the restorations has to be checked under multiple illuminants in order to minimize the metameric effect. Specimen construction and the measurement of their color data is time consuming, but is one-time procedure. In order to avoid repetition of this procedure by every clinician, color data for the most common digital camera models, can be collected and shared, in order to be used with the same polarized filter and white balance card. Special software with artificial intelligence could also help in order for the whole protocol be fully automated.

## Conclusions

Digital evaluation and selection of composite resin system and shade, using a white balance reference card assisted by cross polarized photography, could help achieving more predicTable outcomes using the stratification technique with resin composites.
